# Sodium selenite attenuates sepsis-induced cardiac inflammation and oxidative injury by regulating TXN2/TXNIP/NLRP3 signaling and ferroptosis

**DOI:** 10.3389/fphar.2026.1813332

**Published:** 2026-07-01

**Authors:** Lei Zhang, Dandan Zhu, Jian Yu

**Affiliations:** Department of ICU, Second Affiliated Hospital of Dalian Medical University, Dalian, Liaoning, China

**Keywords:** selenium, sepsis-induced cardiac dysfunction, TXN2/TXNIP, NLRP3, ferroptosis

## Abstract

Septic cardiomyopathy is cardiac dysfunction caused by sepsis and is a common consequence of sepsis. Clinical studies have found patients with sepsis syndrome commonly have low serum selenium (Se) levels, and Se supplementation at doses higher than the daily requirement may reduce mortality; however, the underlying mechanism remains unclear. We found that downregulation of thioredoxin-2 (TXN2)-induced mitochondrial reactive oxygen species (mtROS), which increased inflammatory and oxidative injury in cardiomyocytes, might be a major contributor to septic cardiomyopathy. Mitoquinone mesylate (MitoQ), an antioxidant specifically targeted to mitochondria, increased TXN2 expression and decreased mtROS, thioredoxin-interacting protein (TXNIP, a negative regulator of TXN), nucleotide-binding oligomerization domain (NOD)-like receptor protein-3 (NLRP3) inflammasome activation, and ferroptosis. The results suggest that the TXN2–ferroptosis–NLRP3 pathway may represent a therapeutic target for sepsis-induced cardiac injury. In addition, we found that Se supplementation improved cardiac function and relieved mtROS accumulation and ferroptosis in sepsis-associated cardiac injury by regulating TXN2 and TXNIP/NLRP3 expression.

## Introduction

Sepsis is an abnormal systemic response to what is sometimes an otherwise ordinary infection. Recognized since ancient times as a deadly menace (Faix, 2013), sepsis remains the most common cause of death in critically ill patients worldwide and the most frequent non-cardiac diagnosis in the cardiac intensive care unit, despite significant advances in organized treatment approaches (L'Heureux et al., 2020). The mechanism of sepsis-induced cardiac dysfunction (SIC) involves a variety of molecular pathways. The myocarditis cascade is driven by pathogen-associated molecular patterns (PAMPs) and damage-associated molecular patterns (DAMPs) (Zindel and Kubes, 2020). Other mechanisms are also involved in mediating myocardial injury, including global myocardial ischemia, myocardial depressant substance, inflammatory and adrenergic pathways, altered calcium responsiveness, coronary microvascular dysfunction, and impaired myocardial recovery (Kakihana et al., 2016). Nevertheless, further exploration of the pathophysiology of sepsis-induced cardiomyopathy will provide a basis for the treatment of SIC.

Multiple highly adenosine triphosphate (ATP)-consuming functions are downregulated to conserve energy during sepsis (Peerapornratana et al., 2019). It is well known that sepsis induces significant mitochondrial injury and elevates mitochondrial reactive oxygen species (mtROS) production (Kuang et al., 2024). Mitochondria are responsible for ATP production within the cell and are also a major source of ROS generation (Gheorghiu and Badiu, 2020). A functional mitochondrial pool may confer protection through a myriad of mechanisms, and it is clear that the restoration of oxidative phosphorylation for substrate processing can occur only if functional mitochondria are available. Therefore, improving mitochondrial function may be a critical mechanism for the treatment of SIC.

Mitochondrial ROS (mtROS), one of the most critical pro-inflammatory signals, can be scavenged by the mitochondrial thioredoxin antioxidant system, which compresses thioredoxin-2 (TXN2). In support of a critical role for TXN2 in regulating inflammation, TXN2 suppresses the activation of NLR family pyrin domain-containing 3 (NLRP3) inflammasome (Huang et al., 2022). TXN is a thiol-disulfide oxidoreductase that plays a key role in maintaining redox homeostasis (Gheorghiu and Badiu, 2020). TXN1 and TXN2 are primarily localized in the cytoplasm and mitochondria, respectively, whereas TXN3 is expressed exclusively in the testis (Huang et al., 2022). The activity of TXN2 can be inhibited by thioredoxin-interacting protein (TXNIP), a natural inhibitor of TXN. Activation of the NLRP3/IL-1β pathway via TXN/TXNIP following mtROS overproduction plays a key role in inflammation. The mitochondrial-specific TXN2 antioxidant pathway reduces target proteins and scavenges mtROS by catalyzing thiol-disulfide exchange reactions (Lu and Holmgren, 2014). However, the pathological role of TXN2 remains poorly understood. Previous studies have shown that loss of TXN2 can lead to spontaneous dilated cardiomyopathy and that TXN2 maintains cardiac function by inhibiting mtROS generation. In addition, mitochondria possess their own glutathione pool. Since glutathione (GSH) is synthesized in the cytoplasm, it must be transported into mitochondria (Jastrząb and Skrzydlewska, 2021). A recent study found that TXN2 is associated with glutathione peroxidase-4 (GPX4), an established negative regulator of ferroptosis (Bjørklund et al., 2021). However, the role of TXN2 and its relationship with the ferroptosis regulator GPX4 in SIC has not yet been investigated.

Selenium (Se) is a metalloid with properties similar to those of sulfur. The concentration of Se in soil varies according to type, texture, organic matter content, and rainfall. Its assimilation by plants is influenced by the physicochemical properties of the soil (including redox status, pH, and microbial activity) (Huang et al., 2020). The presence of Se in the atmosphere is associated with both natural and anthropogenic activities. Several studies have found that Se can preserve mitochondrial function and stimulate mitochondrial biogenesis (Mehdi et al., 2013). Several studies found that selenium levels were below the reference range at all measurement periods and suggested that reduced basal selenium levels in patients with sepsis and systemic inflammatory response syndrome were associated with increased mortality (Mehdi et al., 2013; Akyuva et al., 2021). Additionally, selenocysteine is an essential component of TXN reductase, the flavoenzyme responsible for the reduction of TXN. Selenium supplementation in the culture medium increases TXN reductase activity, partly through an increase in TXN reductase protein expression and primarily through an increase in the specific activity of the enzyme (Rusetskaya et al., 1997). Therefore, appropriate Se supplementation may represent a potential protective strategy against SIC. However, the underlying mechanisms of Se-mediated protection remain unclear.

Therefore, the aim of this study was to explore the potential link between TXN2 and GPX4 in the induction of ferroptosis and investigate the potential protective effects of Se supplementation in SIC.

## Methods

### Chemicals and reagents

Sodium selenite (Na_2_SeO_3_, SS, 90% pure, 71950, CAS: 10102-18-8) was purchased from Sigma-Aldrich. TXN2-recombinant protein (HY-P73431) and mitoquinone mesylate (MitoQ, HY-100116A) were both purchased from MedChemExpress (MCE).

### Bioinformatic analysis

The sepsis gene expression dataset GSE100159 was retrieved from the U.S. National Center for Biotechnology Information Gene Expression Omnibus (GEO) dataset (https://www.ncbi.nlm.nih.gov/geo/). This dataset contains peripheral blood gene expression data from 18 patients with sepsis and 12 healthy controls. Differential expression analysis was performed using the limma package in R; although the DESeq2 package is commonly used for differential expression analysis of RNA-seq data, it was not applied in this study because GSE100159 is a microarray dataset. The sepsis group was compared with the healthy control group to identify differentially expressed genes (DEGs). DEGs were screened using a significance threshold of *p* < 0.05 and |log_2_ foldchange| > 1. Functional enrichment analysis of the identified DEGs was conducted using the Database for Annotation, Visualization, and Integrated Discovery (DAVID). Gene Ontology (GO) enrichment analysis was performed to evaluate cellular components (CCs), molecular functions (MFs), and biological processes (BPs) associated with the DEGs. Raw expression data were subjected to background correction, normalization, and logarithmic transformation prior to analysis.

### Animals and treatment

Seven-week-old specific pathogen-free (SPF) male C57BL/6 mice were purchased from Liaoning Changsheng Biotechnology (Shenyang, China) and house individually cages with free access to food and water for 1 week.

To investigate the expression of TXN2 in the SIC model, twelve C57BL/6 mice were randomly assigned to either the control group or the cecal ligation and puncture (CLP) group. To evaluate the role of mtROS and the protective effects of SS in the SIC model, mice were treated with MitoQ (5 mg/kg) for two consecutive days or administered SS orally at a dose of 0.5 mg/kg BW 24 h before surgery (Han et al., 2018; Ala et al., 2022). The mice were divided into four groups, each consisting of seven animals: control group, MitoQ or SS group, CLP group, and CLP + MitoQ or CLP + SS group. All investigators were blinded during outcome assessment. Twenty-four hours after surgery, the mice were anesthetized with 1.5% isoflurane by inhalation and then euthanized and dissected. The hearts were collected for further analysis.

### Echocardiography

Echocardiography measurements were performed using a 30-MHz transducer (Vevo 2100, VisualSonics Inc., Canada), as previously described (Rusetskaya et al., 1997). The mice were anesthetized, and heart rates were maintained at approximately 400 beats/min. M-mode images were obtained from the left parasternal long-axis view of the left ventricle at the level of the papillary muscles. Ejection fraction (EF%) and fractional shortening (FS%) were assessed from the M-mode tracings.

### Histological analysis, immunohistochemistry, and immunofluorescence

Heart tissues were fixed in 4% paraformaldehyde, embedded in paraffin, and sectioned at a thickness of 4 μm. Hematoxylin and eosin (H&E), dihydroethidium (DHE), and Masson’s trichrome staining were performed to assess pathological changes, ROS production, and collagen deposition in the heart tissues. For immunohistochemical staining, heart sections were incubated with an anti-TXN2 antibody (Cusabio, CSB-PA857458ESR2HU, 1:150) at 4 °C overnight. The following day, the sections were washed, incubated with peroxidase-conjugated secondary antibodies (Zhongshan Jinqiao, China), and developed using diaminobenzidine (DAB, Zhongshan Jinqiao, China) to produce a brown reaction product. For immunofluorescence staining, sections were incubated with an anti-CD68 antibody (Arigo, ARG23387, 1:200) or an α-SMA antibody (Proteintech, 14395-1-AP, 1:250) at 4 °C overnight. The following day, the sections were washed and incubated with donkey anti-mice FITC-conjugated secondary antibody (green, ABclonal, AS042, 1:200) at room temperature for 30 min. After washing, the sections were counterstained with 4′,6-diamino-2-phenylindole (DAPI). Images were acquired using a Nikon Eclipse Microscope equipped with a digital camera (Nikon, Tokyo, Japan).

### Measurements of the levels of glutathione, malondialdehyde, superoxide dismutase, and lactate dehydrogenase detection

According to the manufacturer’s instructions, commercial assay kits for malondialdehyde (MDA), GSH, and superoxide dismutase (SOD) (A003-1-2, A006-2-1, and A001-3-2, respectively; Nanjing Jiancheng) were used to measure the levels of MDA, GSH, and SOD in heart tissues. According to the manufacturer’s protocol, serum lactate dehydrogenase levels were determined using an LDH assay kit (A020-2-2, Nanjing Jiancheng).

### Thioredoxin reductase activity assay

Thioredoxin reductase (TrxR) catalyzes the reduction of its substrate, leading to the reduction of a chromogenic agent and the generation of a characteristic absorption peak at 412 nm. According to the manufacturer’s instructions, TrxR activity in heart tissues and cells was measured using a TrxR activity assay kit (E-BC-K548-M, Elabscience).

### Cell culture, viability analysis, and treatment

AC-16 cells were purchased from Procell (Wuhan, China). The cells were cultured in selenium-free DMEM/F12 media (Gibco Thermo Fisher, Carlsbad, CA, USA) supplemented with 10% FBS (HyClone, Logan, UT, United States) and 1% penicillin/streptomycin (Solarbio, Beijing, China) in a humidified incubator containing 95% air and 5% CO_2_ at 37 °C. For selenium supplementation, cells were pretreated with SS (100 nM) for 6 h and subsequently stimulated with LPS (1 μg/mL) for 24 h (Wajner et al., 2015).

To investigate the role of TXN2 in SIC, AC-16 cells were treated with either control or recombinant human TXN protein (10 μM, HY-P73431, MCE) for 6 h, followed by treatment with LPS (1 μg/mL) for 24 h before further experiments (Kamoun et al., 2015; Qayyum et al., 2021).

### Analysis of mtROS

Mito-SOX Red (Invitrogen) staining was used to assess mtROS levels in heart sections and AC-16 cells. AC-16 cells (1 × 10^6^ cells/well) were cultured in 24-well plates, pre-treated with SS (100 nM) for 6 h, and then stimulated with LPS for 24 h. The cells were incubated with Mito-SOX Red probe at a final concentration of 5 μM at 37 °C in the dark for 10 min. After staining, the cells were washed thoroughly with PBS and observed under a microscope. Images were acquired using a Nikon Eclipse Microscope equipped with a digital camera (Nikon, Tokyo, Japan).

### Western blot assay

Total proteins were extracted from snap-frozen heart tissues or cultured cells. Protein extraction was performed using centrifuge tubes (Guangzhou Jet Bio-Filtration Co., Ltd.) and a protein extraction kit (KeyGEN BioTECH, KGP250) according to manufacturer’s instructions. Protein lysates (35 μg per sample) were separated by electrophoresis on 10%–12% SDS-PAGE gels and subsequently transferred onto polyvinylidene difluoride (PVDF) membranes. The membranes were incubated overnight at 4 °C with the appropriate primary antibodies, followed by incubation with goat anti-rabbit or goat anti-mouse secondary antibodies (Sino Biological Inc.). Protein bands were visualized using an ECL Plus chemiluminescence detection system. The following primary antibodies were used: anti-TXN2 (Cusabio, CSB-PA025366GA01HU, 1:500), anti-TXNIP (Cell Signaling Technology, #14715, 1:800), anti-NLRP3 (Proteintech, 30109-1-AP, 1:1000), anti-ACSL4 (Proteintech, 22401-1-AP, 1:800), anti-SLC7A11 (Proteintech, 26864-1-AP, 1:800), and anti-GPX4 (Cusabio, CSB-RA094425A0HU, 1:500). Densitometry analysis was performed using ImageJ software, with β-actin (Bioss Antibodies, bsm-33036M, 1:2500) used as the internal loading control.

### Real-time PCR assay

Total RNA was extracted from fresh heart tissues and cultured cells using TRIzol reagent (Invitrogen, New York) according to the manufacturer’s instructions. First-strand cDNA was synthesized from 2 μg of total RNA using a Superscript II Kit (TAKARA, Japan). All primers were synthesized by Sangon Biotech Corporation (Shanghai, China). The primer sequences are shown in Table 1.

**TABLE 1:** Primers used for RT-PCR.

Gene	Forward primer (5′-3′)	Reverse primer (5′-3′)
Interleukin-1β (IL-1β)	TGC​CAC​CTT​TTG​ACA​GTG​ATG	TGA​TGT​GCT​GCT​GCG​AGA​TT
IL-18	TCT​TCA​TTG​ACC​AAG​GAA​ATC​GG	TCC​GGG​GTG​CAT​TAT​CTC​TAC
Collagen I (Col I)	GAG​TAC​TGG​ATC​GAC​CCT​AAC​CA	GAC​GGC​TGA​GTA​GGG​AAC​ACA
Col III	TCC​CCT​GGA​ATC​TGT​GAA​TC	TGA​GTC​GAA​TTG​GGG​AGA​AT
β-actin	GTG​ACG​TTG​ACA​TCC​GTA​AAG​A	GCC​GGA​CTC​ATC​GTA​CTC​C

### Statistics

All data in this study are presented as the mean ± standard deviation (SD). Statistical analysis was performed using GraphPad Prism 9 software. First, the normality of the data was tested. For normally distributed data, an independent t-test was used to determine statistical differences between two groups; for non-normally distributed data, the Mann–Whitney U test was applied. One-way analysis of variance (ANOVA) was used to compare differences among three or more groups with one independent variable, while two-way ANOVA was employed to analyze the effects of two independent variables and their interaction on the dependent variable. A *p-*value <0.05 was considered statistically significant.

## Results

### TXN2 was decreased in sepsis patients’ peripheral blood and mice’s hearts

**FIGURE 1:**
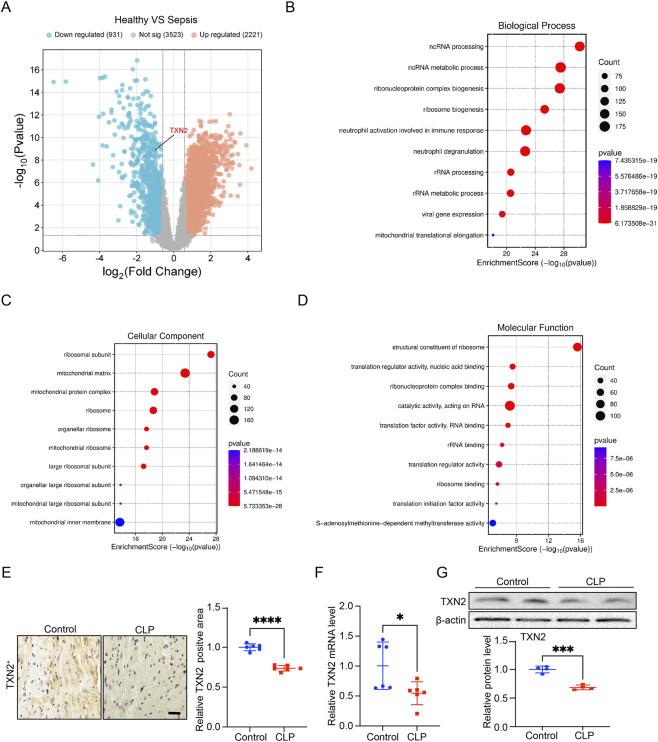
TXN2 expression in the peripheral blood of patients with sepsis and in the hearts of mice. **(A)** Volcano plot illustrating gene expression and TXN2 expression. **(B–D)** GO enrichment analysis showing cellular component (CC), molecular function (MF), and biological process (BP). **(E)** Immunohistochemical staining of heart sections using an anti-TXN2 antibody (n = 6). **(F)** RT-PCR analysis of TXN2 mRNA levels (n = 6). **(G)** Western blot analysis of TXN2 protein expression (n = 4). The results are expressed as the mean ± SD; *p < 0.05, ***p < 0.001, and ****p < 0.0001.

Recent evidence has increasingly implicated TXN2 in sepsis-induced cardiac injury. Analysis of inflammatory gene expression using volcano plots showed that TXN2 expression was significantly decreased in the peripheral blood of patients with sepsis compared with that of healthy controls (Figure 1A). GO enrichment analysis indicated that mitochondrial-related pathways were significantly enriched in the CC, BP, and MF categories (Figures 1B–D). The expression of TXN2 in the cardiac tissues of septic mice was evaluated by immunohistochemical staining, RT-PCR, and Western blot analysis. As shown in Figure 1, compared with the control group, both TXN2 protein and mRNA expression levels were significantly downregulated in the cardiac tissues of septic mice (Figures 1E–G). These findings suggest that decreased expression of TXN2 may contribute to the pathogenesis of SIC.

### Treatment with MitoQ reversed cardiac dysfunction in the hearts of septic mice

**FIGURE 2:**
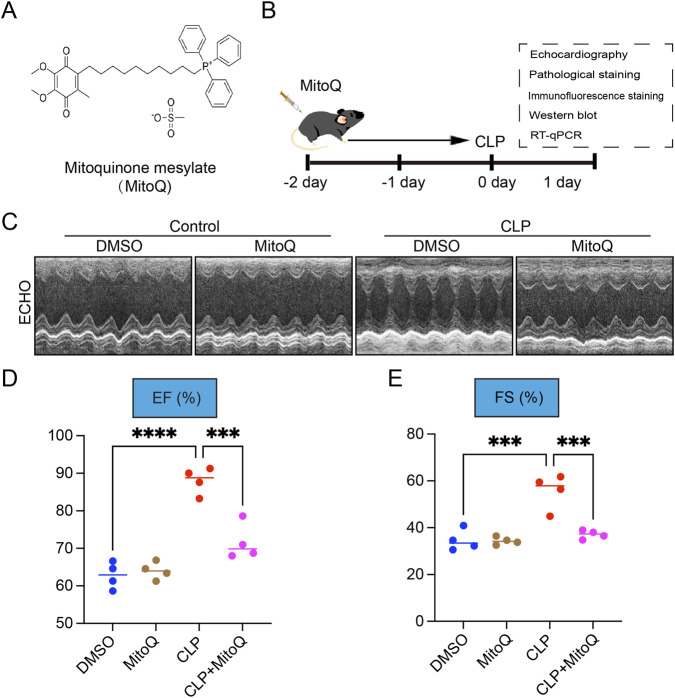
MitoQ improves cardiac dysfunction in septic mice. **(A)** Structural formula of MitoQ. **(B)** Schematic of MitoQ (5 mg/kg for two consecutive days before surgery) to counteract sepsis-induced myocardial injury and experimental design. **(C–E)** Assessment of cardiac function (n = 4). The results are expressed as the mean ± SD; ***p < 0.001 and ****p < 0.0001.

To investigate the role of mitochondrial oxidative stress in SIC, MitoQ, an orally bioavailable mitochondrial-targeted antioxidant, was administered in the septic mouse model. As shown in Figure 2A, the chemical structure of MitoQ is presented. A schematic illustration of the experimental design, including MitoQ administration (5 mg/kg for two consecutive days before surgery) to counteract sepsis-induced myocardial injury, is shown in Figure 2B. Key evaluations included echocardiography, pathological staining, Immunofluorescence staining, Western blotting, and RT-qPCR analysis. Echocardiographic assessment demonstrated that sepsis significantly reduced EF% and FS% compared with the control group. Following MitoQ treatment, both EF% and FS% were markedly improved and restored toward baseline levels (Figures 2C–E).

### MitoQ relieved morphological injury in the hearts of septic mice

H&E staining revealed significant disruption of myocardial tissue architecture in CLP-induced septic mice compared with the control group. Cardiomyocytes exhibited disorganized alignment, accompanied by prominent intracytoplasmic vacuolar degeneration and other morphological abnormalities. Increased proliferation of loose connective tissue was also observed, further contributing to structural disorganization. MitoQ treatment markedly alleviated these sepsis-induced histopathological changes in cardiomyocytes injuries (Figure 2A). Similarly, cardiac LDH levels were significantly reduced in MitoQ-treated septic mice compared with untreated septic mice (Figure 2B). CD68 immunofluorescence staining showed that, compared with the control group, the CLP group exhibited a higher number of CD68-positive macrophages in heart tissues, whereas treatment with MitoQ significantly reduced CD68-positive cell infiltration (Figures 2C,D). Furthermore, Masson’s trichrome staining and α-SMA immunofluorescence staining demonstrated that fibrosis was markedly increased in septic mice compared with controls; these fibrotic changes were substantially attenuated following MitoQ administration (Figures 2E–G). RT-qPCR analysis demonstrated that the mRNA expression levels of inflammatory cytokines (IL-1β and IL-18) and fibrotic-related genes (Col I and Col III) were increased in septic mice compared with the control group. MitoQ treatment significantly reduced the expression of these inflammatory and fibrotic markers (Figures 2H,I).

### MitoQ alleviated ROS accumulation and ferroptosis and NLRP3 inflammasome activation in the hearts of septic mice

**FIGURE 3:**
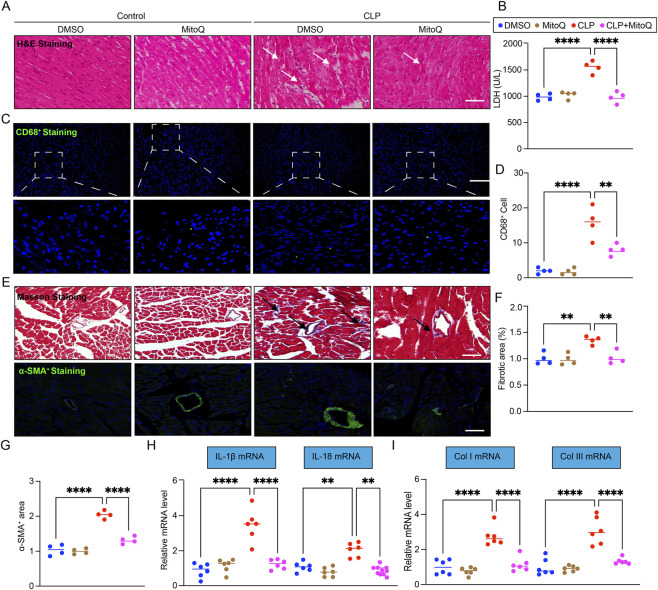
MitoQ rescues sepsis-induced cardiac inflammation and fibrosis. **(A)** H&E staining of heart tissues from each group (scale bar = 50 μm). **(B)** Serum LDH levels in each group (n = 4). **(C,D)** Immunofluorescence staining of CD68 (n = 4, scale bar = 50 μm); quantification of CD68^+^ cells (right, n = 4). **(E–G)** Masson’s trichrome and α-SMA immunofluorescence staining (n = 4, scale bar = 50 μm), with quantification of α-SMA^+^ area. **(H,I)** RT-PCR analysis of IL-1β, IL-18, collagen I, and collagen III mRNA levels (n = 6). The results are expressed as the mean ± SD. **p < 0.01, ***p < 0.001, and ****p < 0.0001.

To further investigate the role of ROS in sepsis-induced cardiac injury, ROS accumulation in heart tissues was evaluated. As shown in Figures 3A,B, DHE staining was performed to assess ROS levels. The results indicated a significant increase in ROS accumulation in the cardiac tissues of septic mice compared with the control group. MitoQ treatment markedly attenuated this increase. To further assess oxidative stress, the levels of MDA, SOD, and GSH were measured in heart tissues. Compared with the control group, septic mice exhibited increased MDA levels and decreased SOD and GSH levels. These changes were significantly ameliorated by MitoQ treatment (Figures 3C–E). The protein expression levels of TXN2, SLC7A11, and GPX4 were examined (Figures 3F,G); the expression of TXN2, SLC7A11, and GPX4 was decreased in septic heart tissues. MitoQ administration upregulated the expression of these proteins. Moreover, the expression levels of TXNIP, NLRP3, and ACSL4 were evaluated (Figures 3F,G). The upregulation of TXNIP, NLRP3, and ACSL4 observed in the septic group was suppressed following MitoQ treatment. These results suggest that TXN2 may regulate ROS accumulation, thereby reducing NLRP3 inflammasome activation and ferroptosis. This mechanism may underlie the protective effects of Se against SIC.

### Upregulation of TXN2 decreased mtROS, ferroptosis, and NLRP3 inflammasome activation in LPS-treated AC-16 cells

**FIGURE 4:**
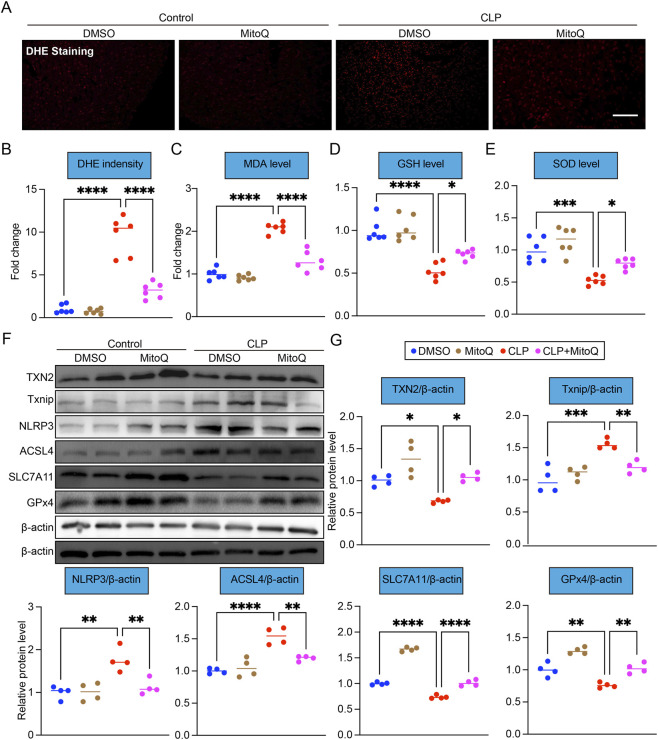
MitoQ reduces oxidative stress and ferroptosis by regulating TXN2–TXNIP–NLRP3. **(A)** DHE staining of heart sections (n = 4, scale bar = 50 μm). **(B)** Quantification of DHE intensity (n = 4). **(C–E)** Levels of MDA, GSH, and SOD. **(F)** Immunoblotting analysis of TXN2, TXNIP, NLRP3, ACSL4, SLC7A11, GPX4, and β-actin (n = 4). **(G)** Relative protein levels of each blot (n = 4). The results are expressed as the mean ± SD. *p < 0.05, **p < 0.01, and ****p < 0.0001.

**FIGURE 5:**
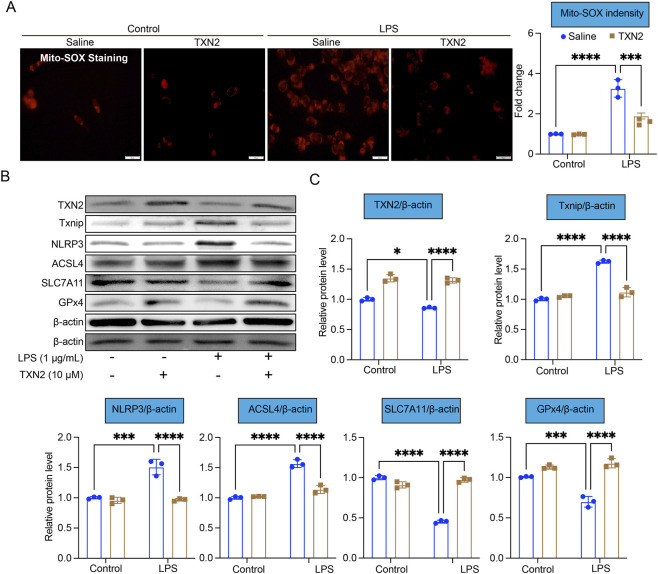
Effects of MitoQ on TXN2-mediated protection against mtROS and ferroptosis. **(A)** Mito-SOX staining of mtROS (left, n = 3, scale bar = 20 μm) and quantification of Mito-SOX intensity (right, n = 3). **(B)** Immunoblotting analysis of TXN2, TXNIP, NLRP3, ACSL4, SLC7A11, GPX4, and β-actin (n = 3). **(C)** Relative protein levels of each blot (n = 3). The results are expressed as the mean ± SD. *p < 0.05, ***p < 0.001, and ****p < 0.00001.

To further explore the regulatory role of TXN2 in SIC, AC-16 cells were pretreated with recombinant human TXN protein for 6 h and then treated with LPS for 24 h. As shown in Figure 5A, Mito-SOX staining showed that TXN2 treatment significantly reduced mtROS levels in LPS-treated cells. Western blot analysis revealed that TXN2 protein treatment increased TXN2 expression in LPS-treated AC-16 cells. The expression levels of SLC7A11 and GPX4 were upregulated, whereas the expression levels of TXNIP, NLRP3, and ACSL4 were decreased after TXN2 administration (Figures 4B,C).

### SS supplementation alleviated sepsis-induced cardiac dysfunction and morphological injury in the hearts of septic mice

**FIGURE 6:**
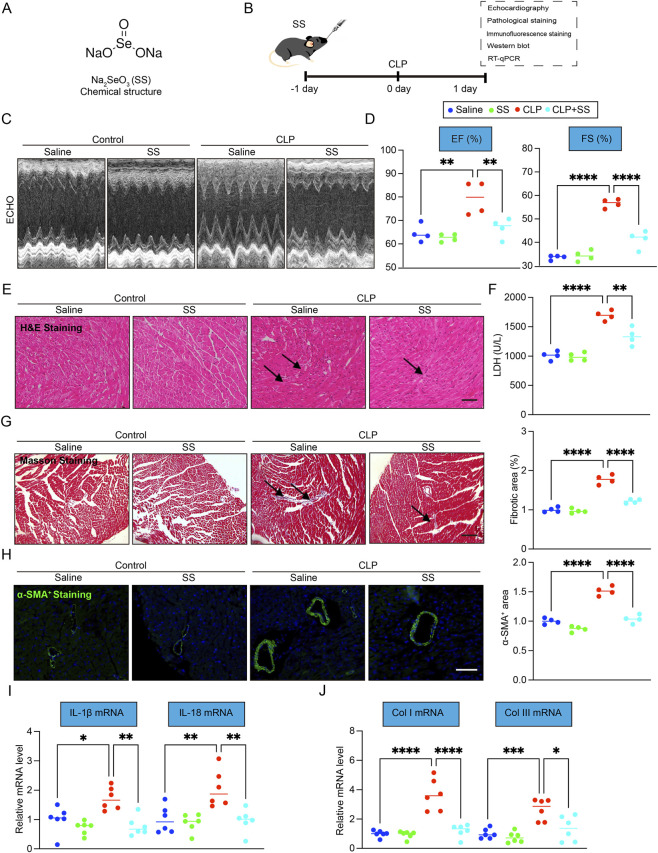
SS improves cardiac dysfunction and pathological injury in septic mice. **(A)** Structural formula of SS. **(B)** Schematic of SS (0.5 mg/kg BW, orally, 24 h before surgery) to counteract sepsis-induced myocardial injury and experimental design. **(C,D)** Assessment of cardiac function (n = 4). **(E)** H&E staining of heart tissues from each group (scale bar = 20 μm). **(F)** Serum LDH levels in each group (n = 4). **(G)** Masson’s trichrome staining (right, n = 4, scale bar = 20 μm) and quantification of fibrotic area (left, n = 4). **(H)** α-SMA immunofluorescence staining (right, n = 4, scale bar = 50 μm) and quantification of α-SMA^+^ area (left, n = 4). **(I,J)** RT-PCR analysis of IL-1β, IL-18, collagen I, and collagen III mRNA levels (n = 6). The results are expressed as the mean ± SD. *p < 0.05, **p < 0.01, ***p < 0.001, and ****p < 0.0001.

The chemical structure of SS is shown in Figure 6A. A schematic illustration of the experimental design, including SS administration (0.5 mg/kg BW, orally, 24 h before surgery) to counteract sepsis-induced myocardial injury, is presented in Figure 6B. Key evaluations included echocardiography, pathological staining, immunofluorescence staining, Western blot analysis, and RT-qPCR (Figure 6B). M-mode echocardiography was used to assess cardiac function in each group (Figures 5C,D). EF% and FS% were decreased in septic mice, and SS administration markedly improved both EF% and FS%, restoring them toward baseline levels (Figures 5C,D). H&E staining showed that treatment with SS significantly alleviated sepsis-induced cardiomyocyte injuries (Figure 5E). Cardiac LDH levels (Figure 6F) were reduced in SS-treated septic mice. Masson’s trichrome staining and α-SMA immunofluorescence were performed to evaluate cardiac fibrosis and collagen deposition. As shown in Figures 5G,H, α-SMA expression and collagen deposition were increased in the hearts of septic mice compared with the control group. These changes were significantly attenuated following SS treatment. mRNA expression levels of inflammatory cytokines (IL-1β and IL-18) and fibrosis-related genes (Col I and Col III) were increased in septic mice. SS administration reduced the expression of these inflammatory and fibrotic markers (Figures 6I,K). These results suggest that SS treatment attenuates cardiac injury in septic mice; however, whether TXN2 is involved in this process remains unclear.

### SS increased TrxR activity and TXN2 expression and decreased ROS accumulation, ferroptosis, and NLRP3 inflammasome activation in sepsis-induced cardiac injury

**FIGURE 7:**
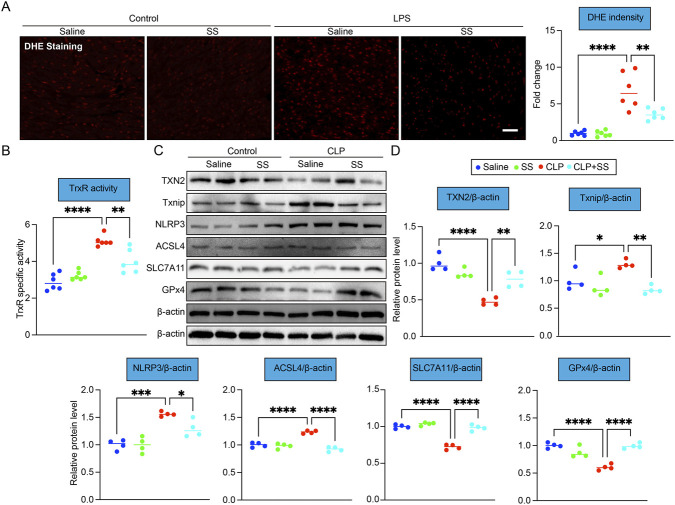
SS reduces oxidative stress and ferroptosis by regulating TXN2–TXNIP–NLRP3. **(A)** DHE staining of heart sections (right, n = 4, scale bar = 20 μm); quantification of DHE intensity (left, n = 4). **(B)** TrxR activity (n = 6). **(C)** Immunoblotting analysis of TXN2, TXNIP, NLRP3, ACSL4, SLC7A11, GPX4, and β-actin (n = 4). **(D)** Relative protein levels of each blot (n = 4). The results are expressed as the mean ± SD. *p < 0.05, **p < 0.01, ***p < 0.001, and ****p < 0.0001.

To investigate the role of TXN2 in sepsis-induced cardiac injury and the effects of SS treatment on TXN2 expression, we first evaluated ROS accumulation in heart tissues. As shown in Figure 7A, DHE staining was performed to assess total superoxide levels. The results demonstrated a significant increase in superoxide accumulation in the hearts of septic mice compared with the control group. SS treatment markedly attenuated this increase. As shown in Figure 7B, TrxR activity decreased in the SIC model group compared with the control group. SS treatment effectively restored TrxR activity. The protein expression levels of TXN2, SLC7A11, and GPX4 were examined (Figures 7C,D). Compared with the control group, the expression of TXN2, SLC7A11, and GPX4 was reduced in septic heart tissues. SS administration increased the expression of these proteins. Moreover, we examined the expression levels of TXNIP, NLRP3, and ACSL4 (Figures 7C,D). The elevated expression of TXNIP, NLRP3, and ACSL4 in the septic group was suppressed following SS treatment. These results suggest that SS may protect against sepsis-induced ferroptosis and NLRP3 inflammasome by upregulating TXN2 expression and reducing mtROS accumulation.

### Se alleviated the downregulation of TrxR activity and TXN2 expression and attenuated mtROS accumulation, ferroptosis, and NLRP3 inflammasome activation in LPS-treated AC-16 cells

**FIGURE 8:**
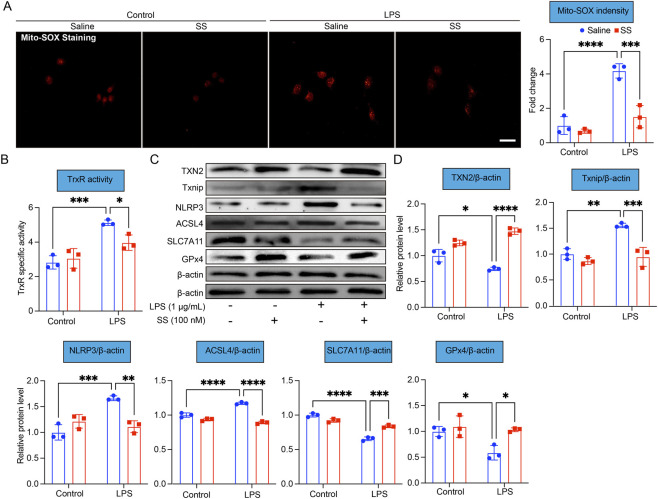
Effects of SS on TXN2-mediated protection against mtROS and ferroptosis. **(A)** Mito-SOX staining of mtROS (left, n = 3, scale bar = 20 μm) and quantification of Mito-SOX intensity (right, n = 3). **(B)** TrxR activity (n = 3). **(C)** Immunoblotting analysis of TXN2, TXNIP, NLRP3, ACSL4, SLC7A11, GPX4, and β-actin (n = 3). **(D)** Relative protein levels of each blot (n = 3). The results are expressed as the mean ± SD. *p < 0.05, **p < 0.01, ***p < 0.001, and ****p < 0.0001.

An *in vitro* study was conducted to further investigate the protective effects of Se supplementation (100 nM) in LPS-treated AC-16 cells. Mito-SOX staining was performed to assess mtROS levels. As shown in Figure 8A, mtROS levels were increased in LPS-treated cells compared with the control group. This increase was suppressed by SS treatment. As shown in Figure 8B, compared with the control group, TrxR activity decreased in the LPS group. SS treatment restored TrxR activity in LPS-stimulated AC-16 cells (Figure 8B). Western blot analysis (Figures 8C,D) demonstrated that the expressions of TXN2, SLC7A11, and GPX4 were reduced in LPS-treated cells, whereas the expression levels of TXNIP, NLRP3, and ACSL4 were increased. SS treatment effectively reversed these changes.

## Discussion

Sepsis is a manifestation of the immune and inflammatory response to infection and may lead to multiple organ failure. Although advances in healthcare have improved outcomes in critically ill patients, sepsis remains a leading cause of mortality worldwide (Song et al., 2023). Septic cardiomyopathy is cardiac dysfunction caused by sepsis and is a complication associated with mortality rates of up to 70%. The pathogenesis of septic cardiomyopathy remains incompletely understood; improved understanding of its underlying mechanisms and the identification of potential therapeutic targets may reduce mortality and improve clinical outcomes in patients with sepsis (Gheorghiu and Badiu, 2020). Previous studies have found that high-dose sodium selenite may improve outcomes in sepsis by attenuating oxidative stress. Patients received an initial intravenous loading dose of sodium selenite (1,000 μg), followed by a continuous intravenous infusion of 1,000 μg/day until discharge from the intensive care unit. High-dose sodium selenite administration was associated with improved outcomes in patients with severe sepsis (Bloos et al., 2016). In present study, based on the reduced Se levels in septic patients, we administrated Se as a protective agent to investigate its therapeutic effects. We found that Se increased TrxR activity and upregulated the expression of TXN2, SLC7A11, and GPX4, thereby attenuating sepsis-induced mtROS accumulation, ferroptosis, and NLRP3 inflammasome activation. We used MitoQ, an orally bioavailable mitochondrial-targeted antioxidant, to further confirm the role of TXN2 in sepsis-induced cardiac injury.

As one of the most studied mitochondrial protein, TXN2 is a small redox protein ubiquitously presented in tissues with high metabolic activity, such as the liver, brain, and heart (Zhang et al., 2004). TXN2 is a key mitochondrial redox protein that regulates ROS levels and maintains mitochondrial function (Chen et al., 2019). Studies have shown that excessive ROS triggers the association of NLRP3 with TXNIP, which promotes the activation of NLRP3 inflammasome and maturation of IL-1β and IL-18 (Zhu et al., 2024; Jia et al., 2020; Chen et al., 2024). TXNIP is an important regulator of the cellular redox state. It binds to and inhibits thioredoxin, and TXNIP–TXN2 are involved in critical redox-dependent signal pathways, including NLRP3 inflammasome activation in a redox-dependent manner (Park et al., 2022; Yoshihara et al., 2014). In our study, we found that sepsis induced a decrease in TXN2 expression and activated the TXNIP–NLRP3 pathway, which contributed to ROS accumulation and the release of the inflammatory cytokines IL-1β and IL-18. Furthermore, we treated cells with recombinant human TXN2 protein. *In vitro*, we observed that upregulation of TXN2 induced mtROS levels and inflammatory injury in AC-16 cells, suggesting that this mechanism may underlie sepsis-induced cardiac injury. These finding indicate that TXN2 may represent a potential therapeutic target for sepsis-induced cardiomyopathy.

Ferroptosis, a newly emerged form of regulated necrotic cell death, has been demonstrated to play an important role in multiple diseases, including cardiac injury (Liang et al., 2022) The canonical regulatory mechanism is mediated by GPX4, the only mammalian enzyme known to catalyze the reduction of phospholipid hydroperoxides into phospholipid alcohols (Seiler et al., 2008). A recent study found that TXN2 is associated with the established negative ferroptosis regulator GPX4 (Roos et al., 2020). In our study, we found that upregulation of TrxR activity and TXN2 may decrease total superoxide levels, mtROS, and MDA levels. Lipid peroxidation and mitochondrial damage are key features of ferroptosis. We observed that TXN2 increased SLC7A11 and GPX4 expression. These findings suggest that TXN2 may regulate ferroptosis-related pathways in sepsis-induced cardiac injury.

See is an essential component of the rare amino acid selenocysteine (Sec) and is incorporated at the catalytic site of various selenium-dependent enzymes, such as those in the thioredoxin system, glutathione peroxidase (GPxs), and methionine sulfoxide reductases. These selenoenzymes play important roles in regulating metabolic activity, immune function, antioxidant defense, and intracellular redox regulation and modulation (Xiang et al., 2024; Zhao et al., 2024). The thioredoxin system, composed of TXN, TrxR, and peroxiredoxin (Prx), serves as an important regulator of intracellular reactive oxygen species (ROS) levels and includes the cytosolic mitochondrial TXN2 system (Mehta et al., 2012; Li et al., 2014). However, Se exerts its beneficial or toxic effects largely in a dose-dependent manner. Both selenium deficiency and excess may impair the ability of cardiomyocytes to defend against oxidative stress induced by exogenous stimuli. Importantly, the beneficial biological effects of selenium are limited to a narrow therapeutic window between Se deficiency and overdose, highlighting the importance of its safe administration.

## Data Availability

The original contributions presented in the study are included in the article/supplementary material; further inquiries can be directed to the corresponding author.
